# The association between living alone and depressive symptoms in older adults population: evidence from the China Health and Retirement Longitudinal Study

**DOI:** 10.3389/fpubh.2024.1441006

**Published:** 2024-10-09

**Authors:** Hui Fang, Yingxin Duan, Yinxin Hou, Haoran Chang, Shanju Hu, Ruyi Huang

**Affiliations:** ^1^School of Management, Shandong Second Medical University, Weifang, China; ^2^College of Public Administration and Law, Hunan Agricultural University, Changsha, China

**Keywords:** depressive symptoms, living alone, social activity, adult children’s relationship satisfaction, older adults

## Abstract

**Objective:**

The goal of this research was to reveal the association between living alone and depressive symptoms in older adults. It also aims to explore the mediating role of social activity and adult children’s relationship satisfaction. Ultimately, the study seeks to add to the body of knowledge for lowering the risk of depression among older people and promoting positive aging.

**Methods:**

Based on information from the 2020 China Health and Retirement Longitudinal Study, the ordinary least square (OLS) regression model and propensity score matching (PSM) were used to investigate the association between living alone and depressive symptoms in older adults and to explore possible heterogeneity in different groups. Utilizing the SPSS PROCESS macro application, the mediation model was constructed. The significance of the mediation effect was investigated using the Bootstrap technique.

**Results:**

The average level of depressive symptoms of older people living alone (10.55 ± 6.962) was higher than that of older people who do not live alone (8.80 ± 6.363). The baseline regression analysis revealed a significant connection between living alone and the depressive symptoms of older people (*β* = 0.842, *p* < 0.001). The association between living alone and the level of depressive symptoms was significantly higher in those aged 60–74 years (*β* = 1.698, *p* < 0.001) than in those aged 75 and older (*β* = 0.953, *p* < 0.05). The association between living alone and depressive symptoms was significantly higher in rural older adults (*β* = 1.512, *p* < 0.001) than in urban older adults (*β* = 1.141, *p* < 0.001). Between living alone and the level of depressive symptoms experienced by older people, there was a substantial mediation impact on social engagement and adult children’s relationship satisfaction, which contributed to 2.91 and 13.62% of the overall effect.

**Conclusion:**

For older age groups, living alone is associated with higher levels of depressive symptoms. This effect is stronger in older adults aged 60–74 or rural areas. In older age groups, the association between living alone and depressive symptoms is mediated by social activity and adult children’s relationship satisfaction.

## 1 Introduction

The National Bureau of Statistics has released data displaying that the percentage of people aged 65 and above in China’s overall population has risen from 9.1% in 2011 to 15.4% in 2023. The proportion is expected to continue growing, highlighting the increasingly significant issue of population aging. Depression is a common psychiatric condition marked by enduring feelings of sorrow, emptiness, and disinterest, accompanied by somatization and cognitive changes (1). It is predicted that major depression will become the largest factor in the global illness burden by 2030 (2). According to WHO, almost 280 million individuals globally experience depression, which makes up 4.3% of the total illness burden. Among them, 5.7% are aged 60 years or older. Research conducted in several nations indicates an increasing frequency of depression among older adults (3, 4). The incidence of depression in older adults in China witnessed a significant rise from 36.8% in 2011 to 44.5% in 2018, reflecting an increase of around 10% (5). As an important public health problem, depression seriously endangers the health of the population (6). For example, depression increases the risk of cardiovascular disease (7), diabetes (8), and chronic obstructive pulmonary disease (9). In severe cases, depression can even result in mortality, placing a significant cost on both families and society. Therefore, how to lower the depressive symptoms of older people and avoid suffering from depression is one of the key goals of implementing health interventions.

China, deeply influenced by Confucian culture, has a well-established custom of intergenerational cohabitation, where older adults reside with their offspring. Nevertheless, as a result of the aging population, the rise in life expectancy, and the size of families gradually shrank, the percentage of older people living alone has been steadily rising (10). Most older people living alone are not accompanied by their adult children and other family members due to their widowhood or separation. This renders them a vulnerable subset of older people who require particular care and attention (11). Older people living alone are more prone to loneliness and depression, which can potentially result in an increased occurrence of illnesses, including suicide and mortality (12). However, living alone is not always detrimental to the physical and mental health of older people. Research has indicated that older adults who live alone have a decrease in their health burden (13). Additionally, living alone alleviates the life pressure experienced by older women (14), allowing them to have more time and energy to enjoy later life while diminishing their depressive symptoms. This study utilizes the 2020 China Health and Retirement Longitudinal Study (CHARLS) to investigate the association of living alone with depressive symptoms in older adults and to reveal the role of potential factors. The purpose is to formulate targeted measures to lower the risk of depression among older people living alone.

## 2 Literature review

### 2.1 The direct influence of living alone on the depressive symptoms of older people

Depression is a prevalent disease. Long-term depression has significant detrimental effects on the patient’s mood, cognition, body, and behavior. These effects include a lack of interest in their surroundings, reduced contentment with life, and impaired cognitive performance (15). In order to mitigate the detrimental consequences of depression and the ongoing rise in its prevalence, it is vital to comprehend the factors that lead to depression in various demographic groups. Older adults living alone are a vulnerable group among older people and require special attention. They may face a range of problems including shrinking social networks, social isolation, and loss of social roles (16). They have limited access to social support and emotional comfort, intensifying feelings of loneliness and contributing to a deterioration in mental health (17). In particular, older adults who live alone due to the death of a spouse experience extended periods of anxiety and depression to adjust to negative shocks (18). Nevertheless, older people living alone are highly heterogeneous. While some older people living alone may be socially disconnected, others remain actively integrated into society (19).

Therefore, different scholars hold different views on the relationship between living alone and the mental health of older adults. A study conducted in China indicates that living alone is a predictor for identifying a high prevalence of depression in older adults (20). Compared with those living with others, older adults who live alone are at higher risk of feeling lonely (12) and depressed (21). Srivastava’s study revealed that widowed older adults in India who lived alone had a 56% greater chance of experiencing depression compared to those who were married and did not live alone (22). This finding was confirmed by a large sample of research in the UK, which found that living alone was a high-risk factor in developing depression (23). However, living alone did not mean that older adults living alone were any more likely to feel lonely or depressed than those not living alone. Living alone has less of an impact on older adults’ mental and physical wellness because most of them have comparatively strong physical and cognitive abilities (24). A study in South Korea showed that older women in nuclear households were more prone to depressive symptoms than female older people living alone (25). Similarly, a Chinese scholar found that living with children may lead to a decline in the ability of older people to take care of themselves, and may even be a disadvantage that affects their physical and mental health (26). According to the theory of intergenerational conflict, the conflict between family members will lead to greater pressure on older people who do not live alone (27). Some studies have shown that the amount of depression was not significantly affected by whether older people lived alone or with family over many generations, and for older women, living alone actually improved mental health (14).

### 2.2 The mediating role of social activity

The degree of a person’s participation in various social activities, such as volunteer work and recreational activities, was referred to as their social activity (28). This includes the specific activities and how often the individual participates in them. Previous studies have demonstrated that older people who live alone may experience social dislocation or a loss of social cohesiveness (16). Nevertheless, research conducted in Singapore has revealed that 85.6% of older adults who lack social connections do not live alone (29). Within economically advanced nations, older people who live alone pay more attention to the role of friendship, have more friends in their social networks, and have less dependence on their families (30). Older adults living with disabilities who are capable of living alone probably have fewer limitations compared to those who are living with their families. They can engage in social activities that mitigate the negative association of living alone with depressive symptoms (31).

In addition, older adults’ mental health benefits from high-level social activities. The mental health of older people is influenced by neighborhood care, social support, and social networks (32). The theory of social integration holds that individuals maintain strong connections with their families, neighbors, and friends, forming social networks through which older adults’ thoughts or behaviors are controlled or regulated (33). Older adults’ social activity may be stabilized and their subjective well-being raised by engaging in more social activities on a regular and high-quality basis (34). Older adults with the highest degree of social activity exhibited superior mental health compared to other groups (35). Both online and offline social participation contribute to older adults’ development of a feeling of belonging and identity, as well as alleviating their depressed symptoms and negative emotional encounters (36).

### 2.3 The mediating role of adult children’s relationship satisfaction

Social relationships have an essential function in the overall well-being of older adults, with the bond between adult children being the fundamental social connection (37). Adult children’s relationship satisfaction refers to the degree of contentment experienced by older people with regard to their relationships with their offspring. Higher satisfaction means closer relationships between adult children and older adults, and more frequent parent–child contact. Living alone is an important predictor of adult children’s relationship satisfaction (38). A study showed a positive correlation between living with adult children and the subjective well-being of older people compared to those living alone (39). Under the condition of insufficient pension and social welfare system, disadvantaged older people rely heavily on the financial, emotional, and nursing support provided by their adult children to maintain their daily lives. Therefore, living with their adult children is considered one of the most direct types of family support (40). However, older people living alone have limited opportunities to exchange support with their adult children, which in turn affects the intimacy of the parent–child relationship (41). Living alone and lower adult children’s relationship satisfaction are risk factors for social isolation among older adults, leaving them in a state of disrupted interpersonal and social interaction (42). Lower adult children’s relationship satisfaction means that older adults are at risk of falling into family isolation as their expectations of family are not realized (24). Similarly, family closeness can be considered a potential protective factor for depressive symptoms in older adults living alone (43). Older adults with higher satisfaction with their adult children’s relationships are more likely to feel a sense of family belonging, accept help and companionship from their adult children, and reduce the risk of social isolation.

## 3 Theoretical framework

This study utilizes ecosystem theory as a theoretical framework. Ecosystem theory acknowledges that people do not exist independently but interact with and have an impact on the environment in which they live (44). Based on the degree of influence from small to large, ecosystem theory classifies systems into three levels: micro, meso, and macro. The microsystem pertains to the immediate environment in which a person lives. The mesosystem refers to smaller-scale environments like family, friends, neighbors, and other direct contacts. The macrosystem refers to ecosystems that are larger than the meso level, such as the community, social, and cultural environments in which a person lives (45).

Ecosystem theory emphasizes that the health status of a person is related to interacting ecosystems (46). In this study, the term “micro-system” pertains to the living arrangements of older people, specifically whether they live alone or not. The term “meso-system” refers to the older adults’ satisfaction with their adult children’s relationships. The term “macro-system” refers to the older adults’ level of activeness in participating in social activities. Hsu showed that older adults living alone with higher levels of social activeness had autonomous and active lifestyles. They also revealed that pleasant social surroundings and environments with solid family relationships are crucial to reducing depressive symptoms among older adults living alone (24). Ren and Lu agreed that older adults living alone may experience pleasure and pride by engaging in social activities or getting assistance from their children (47). This contributes to maintaining a positive outlook on life and reduces the likelihood of experiencing depressive symptoms.

Based on the above theoretical framework and empirical evidence from the literature, this study proposes the following hypotheses:

*Hypothesis 1*: Living alone has a significant positive correlation with the depressive symptoms of older people.

*Hypothesis 2*: Social activity plays an intermediary role between living alone and the depressive symptoms of older people.

*Hypothesis 3*: Adult children’s relationship satisfaction plays an intermediary role between living alone and the depressive symptoms of older people.

**Figure 1:**
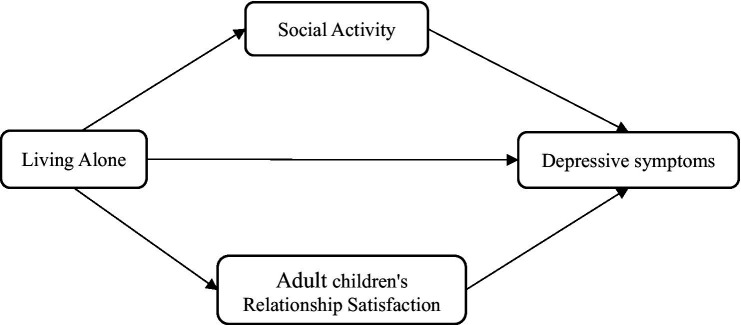
Hypothetical model.

In summary, this study has constructed the model using the hypotheses indicated before, as seen in Figure 1.

## 4 Research design

### 4.1 Data sources

**Figure 2:**
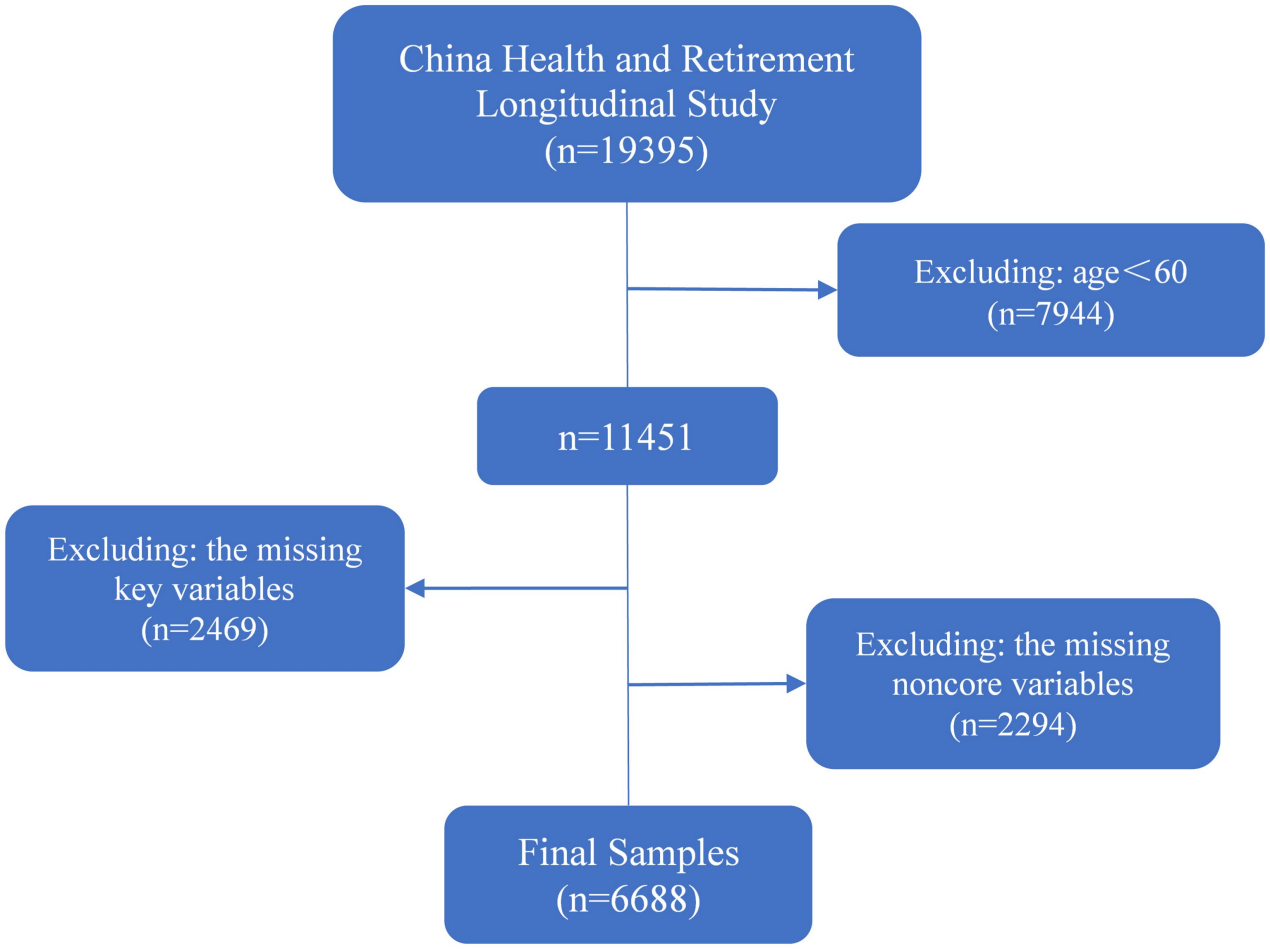
Sample selection flowchart.

Data from the 2020 China Health and Retirement Longitudinal Study served as the basis for this investigation. The survey was a long-term investigation conducted by Peking University’s National Development Research Institute. The participants were individuals above the age of 45 in China. The survey was initially performed in 2011 using the multistage probability proportional to scale (PPS) sampling approach. It spanned 28 provinces and regions of China, with 150 county-level units and 450 village-level units. It had a wide geographical and large sample representation. The 2020 CHARLS survey was the fifth round of tracking survey to be conducted in 2020, which collected fundamental data on individuals and families, health status, cognitive function, work and retirement, household income and expenditure, and epidemiological modules. The data were utilized to conduct a more thorough analysis of China’s aging population. The study included a total of 11,451 individuals aged 60 years and older. Participants with missing essential and non-essential variables were excluded, resulting in a final sample size of 6,688 (see Figure 2).

### 4.2 Variable selection

#### 4.2.1 Dependent variable

##### 4.2.1.1 Depressive symptoms

The CESD-10 is a tool used to assess the depressive symptoms of older adults. It has demonstrated strong validity and reliability in testing with older people in China (48). The CESD-10 questionnaire comprises 10 items, including “I am troubled by some trivial matters” and “I have difficulty in concentrating when doing things,” etc. Eight of the questions are connected to negative emotions, and the remaining two are related to positive emotions. There are four options for each question: little or never (1 day), medium or less frequently (1 ~ 2 days), occasionally or a half times (3 ~ 4 days) and majority of the times (5 ~ 7 days), assigned a value of 0 ~ 3 points. Positive emotional questions are assessed in reverse order. The total value ranges from 0 to 30 points. Greater scores are correspond with higher levels of depressive symptoms in older adults. In this study, the Cronbach’s αcoefficient of the scale is 0.733.

#### 4.2.2 Independent variable

##### 4.2.2.1 Living alone

The length of time spent with older people is obtained from CHARLS’ questions “How long did you live with your spouse or partner in the past year” and “How long did your adult children live with you in the past year.” According to Zheng et al. (20) defined living alone as “older people who had live with their spouses, parents or parent-in-law, offspring, brothers and sisters for less than 11 months in the past year.”

#### 4.2.3 Mechanism variables

##### 4.2.3.1 Social activity

According to CHARLS’ question, “Have you spent the last month participating in any of the following social activities?” determines whether older adults participate in social activities (including going out, volunteering, going to school, etc.). Based on the question “How often did you do these activities in the past month?” determines how frequently older people engage in social activities (almost daily = 3, almost weekly = 2, infrequently = 1). Calculate social activities using the Equation 1:


(1) $$\mathrm{Act}=\overset{N=8}{\underset{i=1}{\sum }}\left(A_{i}\times F_{i}\right)$$

A_i_ indicates whether to participate in social activity plans (yes = 1, no = 0), and F_i_ indicates the frequency of participating in each social activity plan. The numerical range of social activities is 0 ~ 24 points. The more active older adults participate in social activities, the higher the score.

Adult children’s relationship satisfaction. The “Are you pleased with the way you interact with your kids?” survey gauges how satisfied parents are with their relationships with their kids. There are five possible answers which were assigned a value of 1 ~ 5. The higher the assigned value, the more satisfied the bond between aging parents and their offspring.

#### 4.2.4 Control variables

The control variables for this study, as outlined in Table 1, include gender, age, place of residence, education level, marital status, family economic position, self-rated health status, chronic illnesses and ADL. Gong’s approach of measuring *per capita* household consumption is used to determine the economic status of a family (49). The ability of daily activities is measured according to the questionnaire question “Do you have difficulty in dressing, bathing, eating, getting up, going to the toilet, and controlling defecation because of health and memory?.” Each question corresponds to four options: “No difficulty,” “It is difficult but can still be completed,” “It is difficult and needs help” and “It can’t be completed,” assigned a value of 0 ~ 3 points. The range of values in 0 ~ 18 scores. Older adults’ capacity to carry out activities of daily living declines with increasing score.

**Table 1:** Variable definitions.

Variables	Definition and description
Depressive symptoms	Continuous variable
Living alone	1 = yes, 0 = no
Social activity	Continuous variable
Adult children’s relationship satisfaction	1 = totally disgruntled, 2 = not quite gratified, 3 = somewhat gratified, 4 = fairly gratified, 5 = extremely gratified
Gender	1 = male, 0 = female
Age	Continuous variable (age in 2020)
Residence	1 = rural, 0 = urban
Education level	1 = the lowest level of education, 2 = elementary education, 3 = intermediate education, 4 = high education and above
Marital status	1 = married, 0 = else
Family economic position	Continuous variable (Ln(*per capita* household consumption+1))
Self-reported health status	1 = very terrible, 2 = terrible, 3 = fair, 4 = well, 5 = excellent
Chronic illnesses	1 = yes, 0 = no
ADL	Continuous variable

### 4.3 Statistical analysis

#### 4.3.1 Ordinary least square

Because the depressive symptoms in this study are a continuous variable, the ordinary least square approach was chosen for examining the association between living alone and the depressive symptoms of China’s senior population. The model that this paper builds is as follows:


(2) $${\mathrm{depression}}_{i}=\alpha_{0}+\alpha_{1}\mathrm{alon}e_{i}+\gamma_{1}z_{i}+\varepsilon_{i}$$

In Equation 2, the explained variable ${\mathrm{depression}}_{i}$ represents the depressive symptoms of the survey sample; the core independent variable $\mathrm{alon}e_{i}$ represents the living alone situation of the survey sample; $z_{i}$ is the other control variables. $\alpha_{0}$ is the intercept term, $\alpha_{1}$ and $\gamma_{1}$ are the regression coefficients of the corresponding variables and $\varepsilon_{i}$ is the random error term.

#### 4.3.2 Propensity score matching

The Propensity score matching approach, which was originally put forward by Rosenbaum and Rubin, is a better way to handle endogenous problems. Because it can effectively eliminate the mixed bias through a series of matching processes, and partially resolve the issue of selective bias in observational experiments.

This study utilized propensity score matching to assess the effect of living alone on depressive symptoms in older adults, in order to examine the reliability of the findings obtained from the OLS regression model. First of all, this study divides the samples into two groups: the treatment group (living alone) and the control group (not living alone). It matched the samples of each control variable utilizing the Logit model and evaluated the likelihood of the sample individuals entering the treatment group according to the observable characteristics. That is, the propensity score is calculated by the Logit model:


(3) $$P\left(X_{i}\right)=\mathrm{Pr}\left(D_{i}=1|X_{i}\right)=E\left(D_{i}=0|X_{i}\right)$$

In Equation 3, $D_{i}=1$ indicates living alone, $D_{i}=0$ indicates not living alone, and $X_{i}$ is a series of control variables.

Secondly, according to the propensity score choose the appropriate matching technique to align the samples. Diverse matching strategies provide varying variances. In order to ensure the robustness of the results, this research selects nearest neighbor matching (k = 3), radius matching and kernel matching, among which the caliper of radius matching is 0.01. After completing the three matching methods to match the samples, check the balance of the matched control variables in the treatment group and the control group. If there is no significant difference, move on to the following action.

Finally, based on the matched samples, the average treatment impact on the treated (ATT) of older people living alone is calculated:


(4) $$ATT=E\left(Y_{1i}|D=1\right)-E\left(Y_{0i}|D=1\right)$$

In Equation 4, $Y_{1i}$ denotes the depressive symptoms of older adults living alone, and $Y_{0i}$ denotes the depressive symptoms of older adults not living alone.

#### 4.3.3 Test of mediating effects

To investigate the mechanism of the effect of social activeness and adult children’s relationship satisfaction between living alone and depressive symptoms in older adults. This study used the SPSS macro program PROCESS developed by Hayes (50) to construct a mediated effect model. Model 6 examines whether there is a chain mediating effect between social activity and adult children’s relationship satisfaction. Model 4 examines the parallel mediating effect of social activity and adult children’s relationship satisfaction. The significance of the regression coefficient was tested by deviation correction Bootstrap 95% confidence interval and 5,000 iterations of sampling were used. The outcome is significant if there is no zero in the confidence interval.

## 5 Results

### 5.1 Descriptive analysis

The results of descriptive statistics for older adults living alone and non-living alone are shown in Table 2. The results showed that 19.2% of older adults were living alone and 80.8% were non-living alone. The average level of depressive symptoms among older adults who live alone (10.55 ± 6.962) was higher compared to older adults who do not live alone (8.80 ± 6.363) (*p* < 0.001). Social activeness among older adults living alone (1.51 ± 2.017) was also higher than that older adults not living alone (1.31 ± 1.880) (*p* < 0.001). 45.2% of older adults living alone and 47.4% of older adults not living alone reported being extremely satisfied with their adult children’s relationship satisfaction (*p* < 0.001).

**Table 2:** Demographic characteristics of 6,688 samples.

Variable	Overall (*n* = 6,688)	Living alone (*n* = 1,284)	Not living alone (*n* = 5,404)	*p* value
Depressive symptoms	9.14 ± 6.518	10.55 ± 6.962	8.80 ± 6.363	<0.001
Social activity	1.35 ± 1.909	1.51 ± 2.017	1.31 ± 1.880	<0.001
Adult children’s relationship satisfaction				<0.001
Totally disgruntled	106(1.6)	35(2.7)	71(1.3)	
Not quite gratified	260(3.9)	69(5.4)	191(3.5)	
Somewhat gratified	2,782(41.6)	523(40.7)	2,259(41.8)	
Fairly gratified	3,139(46.9)	580(45.2)	2,559(47.4)	
Extremely gratified	401(6.0)	77(6.0)	324(6.0)	
Gender				<0.001
Male	3,408(51.0)	569(44.3)	2,839(52.5)	
Female	3,280(49.0)	715(55.7)	2,565(47.5)	
Age	68.87 ± 6.265	69.73 ± 7.033	68.67 ± 6.052	<0.001
Residence				<0.001
Rural	4,077(61.0)	851(66.3)	3,226(59.7)	
Urban	2,611(39.0)	433(33.7)	2,178(40.3)	
Educational level				0.449
The lowest level of education	3,151(47.1)	651(50.7)	2,500(46.3)	
Elementary education	1,441(21.5)	257(20.0)	1,184(21.9)	
Intermediate education	1,247(18.6)	216(16.8)	1,031(19.1)	
High education and above	849(12.7)	160(12.5)	689(12.7)	
Marital status				<0.001
Married	5,520(82.5)	520(40.5)	5,000(92.5)	
Else	1,168(17.5)	764(59.5)	404(7.5)	
Family economic position	9.42 ± 0.876	9.43 ± 0.998	9.41 ± 0.845	
Self-reported health status				0.771
Very terrible	485(7.3)	100(7.8)	385(7.1)	
Terrible	1,339(20.0)	242(18.8)	1,097(20.3)	
Fair	3,377(50.5)	636(49.5)	2,741(50.7)	
Well	787(11.8)	176(13.7)	611(11.3)	
Excellent	700(10.5)	130(10.1)	570(10.5)	
Chronic illnesses				<0.001
Yes	5,777(86.4)	1,132(88.2)	4,645(86.0)	
No	911(13.6)	152(11.8)	759(14.0)	
ADL	0.50 ± 1.099	0.56 ± 1.159	0.49 ± 1.083	0.001

Depressive symptoms, social activity, age, family economic position, and ADL are continuous variables, so their results are expressed as mean ± standard deviation; adult children’s relationship satisfaction, gender, residence, educational level, marital status, self-reported health status, and chronic illnesses are categorical variables, so their results are expressed as [*n*(%)]; *t*-tests and chi-square tests were, respectively, used to calculate the *p*-values for the continuous variables and categorical variables.

### 5.2 Baseline regression results

Firstly, the association of living alone on the depressive symptoms in older adults was examined using the conventional least squares approach (OLS). Table 3 shows the results of benchmark regression. Model (I) is the benchmark model. It is found that living alone is associated with higher levels of depressive symptoms in older adults. Based on model (I), model (II) and model (III) gradually incorporate controlled variables and intermediate variables. The results also show that living alone can significantly increase the depressive symptoms of older adults. The findings further confirm hypothesis 1. At the statistical significance of 0.001, social activity and adult children’s relationship satisfaction are negatively correlated with depressive symptoms in older adults. This means that when social activity and adult children’s relationship satisfaction increase, the depressive symptoms in older adults decrease. Furthermore, the inclusion of intermediary variables (social activity and satisfaction with adult children’s relationship) in the model (II) leads to a decrease in the direct predictive effect of living alone on depressive symptoms in older adults. It is indicated that there may be an intermediary effect between them, which needs further testing.

**Table 3:** Baseline regression results.

Variable	Model (I)	Model (II)	Model (III)
Living alone	1.745^***^	0.940^***^	0.842^***^
	(0.201)	(0.207)	(0.203)
Gender		−1.422^***^	−1.536^***^
		(0.146)	(0.144)
Age		−0.035^**^	−0.032^**^
		(0.012)	(0.011)
Residence		1.490^***^	1.462^***^
		(0.150)	(0.148)
Educational level		−0.680^***^	−0.681^***^
		(0.071)	(0.070)
Marital status		−0.940^***^	−0.976^***^
		(0.225)	(0.221)
Family economic position		−0.034	−0.020
		(0.082)	(0.081)
Self-reported health status		−1.765^***^	−1.601^***^
		(0.074)	(0.073)
Chronic illnesses		0.564^**^	0.601^**^
		(0.207)	(0.203)
ADL		1.358^***^	1.303^***^
		(0.067)	(0.066)
Social activity			−0.120^***^
			(0.036)
Adult children’s relationship satisfaction			−1.426^***^
			(0.092)
Constant	8.803^***^	17.286^***^	22.179^***^
	(0.088)	(0.954)	(1.261)
*N*	6,688	6,688	6,688
R^2^	0.011	0.274	0.300
Adj-R^2^	0.011	0.272	0.299

***p < 0.001, **p < 0.01.

### 5.3 Heterogeneity test

There may be obvious differences in the depressive symptoms of different older groups living alone. In this study, different ages or residences were divided into groups. The specific results are shown in Table 4.

**Table 4:** Heterogeneity analysis results.

	Age		Residence	
	The younger	The older	Urban	Rural
Living alone	1.698^***^	0.953^*^	1.141^***^	1.512^***^
	(0.198)	(0.378)	(0.280)	(0.222)
Control variables	Yes			
Constant	17.303^***^	17.305^***^	15.382^***^	15.114^***^
	(0.907)	(1.769)	(1.296)	(1.070)
Observations	5,458	1,230	2,611	4,077
Adj-*R*^2^	0.262	0.142	0.244	0.243

***p < 0.001, *p < 0.05.

#### 5.3.1 Age heterogeneity

To analyze the age difference in depressive symptoms of older people living alone, this paper divides older adults into two groups: the younger (60 ~ 74 years old) and the older (≥75 years old) for heterogeneity analysis. The findings indicate that living alone has a positive and significant association with the level of depressive symptoms among older people aged 60–74, and those aged 75 and older. This association between depressive symptoms of older people aged 60–74 is more intense, which is significant at the statistical level of 0.1%. Older adults aged 75 and older were statistically relevant only at the 5% level, so it can be considered that living alone has an age difference in depressive symptoms of older people.

#### 5.3.2 Urban–rural heterogeneity

To analyze the urban–rural differences in the depressive symptoms in older adults living alone, this paper divides older adults into two groups according to their residence, rural and urban, and analyzes the heterogeneity. The findings indicate that the depressive symptoms of older adults in rural and urban areas are significantly positively correlated at the statistical level of 1%. The influence coefficient of living alone on older adults’ level of depression in rural areas is 1.512, while the influence coefficient of living alone on the level of depressive symptoms of older adults in urban areas is 1.141. It is feasible to claim that older adults’ depressive symptoms vary across rural and urban regions.

### 5.4 Robustness tests

#### 5.4.1 Model replacement

To enhance the reliability of research findings, researchers often opt for either variable replacement or model replacement methods for testing purposes (see Table 5). This study employs the substitution statistical method to assess the reliability of the findings. The robustness of the model is evaluated by comparing the direction, magnitude, and significance of the estimated coefficients of the primary explanatory variables. According to Andresen’s research, the total score of the CESD-10 scale is ≥10, which means that older adults suffer from depression (51). Accordingly, if the level of depressive symptoms of older people is less than 10 points, they assign 0 points. Otherwise, it is assign 1 point. The binary logistic regression model is used to replace the OLS regression model for the robustness test.

**Table 5:** Robustness test results based on the model substitution method.

Variable	*β*	S. E.	Wald	*P*	95%CI	
					LLCI	ULCI
Living alone	0.322	0.083	15.034	<0.001	1.173	1.624
Control variables	Yes					
Observations	6,688					
*R* ^2^	0.188					

The regression’s results show that living alone is associated with higher levels of depressive symptoms in older adults at the statistical level of 0.001. The direction and significance of the coefficient after replacing the model are essentially corresponding to the OLS regression model, which shows that the model has good robustness.

#### 5.4.2 Propensity score matching

The traditional linear regression model may have individual self-selection behavior, resulting in biased estimation results. To assess the robustness of OLS regression model findings and deal with endogeneity issues in model design and sample selection. The propensity score matching method is employed in this study to test the empirical results. Multiple methods of nearest neighbor matching (k = 3), radius matching, and kernel matching are adopted for sample matching, respectively. The balancing test has been passed by all three matching strategies. Due to the limited space, this paper only gives the balance test results of kernel matching, as shown in Table 6. Before matching, the variations between the treatment group and the control group were significant at the statistical level of 1% or 5%, except for the variables of family economic status and self-rated health status, which fully showed that there was a self-selection effect in living alone. Taking kernel matching as an example, it is discovered that the statistical value of the test is greater than 0.05 and that the deviation ratio of covariates before and after matching is greatly reduced. It can be considered that the heterogeneity between the treatment group and the control group was effectively weakened, and the matching effect was good. So it passes the balance test.

**Table 6:** Balance test.

Variable	Sample	Mean		S. E.	%bias	*t*-test	
		Treated	Control			*t*	*p*
Age	U	69.731	68.671	16.1	65.8	5.46	0.000
	M	69.731	70.093	−5.5		−1.31	0.190
Residence	U	0.663	0.597	13.7	66.2	4.35	0.000
	M	0.663	0.641	4.6		1.18	0.237
Educational level	U	1.910	1.983	−6.7	32.6	−2.17	0.030
	M	1.910	1.861	4.5		1.17	0.243
Marital status	U	0.405	0.925	−132.1	100.0	−52.42	0.000
	M	0.405	0.405	0.0		0.00	1.000
Family economic position	U	9.429	9.412	1.8	20.9	0.62	0.534
	M	9.429	9.443	−1.4		−0.36	0.717
Self-reported health status	U	2.995	2.979	1.7	6.8	0.53	0.593
	M	2.995	2.980	1.5		0.38	0.703
Chronic illnesses	U	0.882	0.860	6.6	78.8	2.07	0.038
	M	0.882	0.877	1.4		0.36	0.715
ADL	U	0.562	0.487	6.7	37.4	2.21	0.027
	M	0.562	0.610	−4.2		−1.00	0.319

The mismatched subsample is denoted by “U”; the matched subsample is denoted by “M”; % bias denotes mean standardized difference in percentage; the balancing test results here are from Kernel matching of the whole sample.

**Figure 3:**
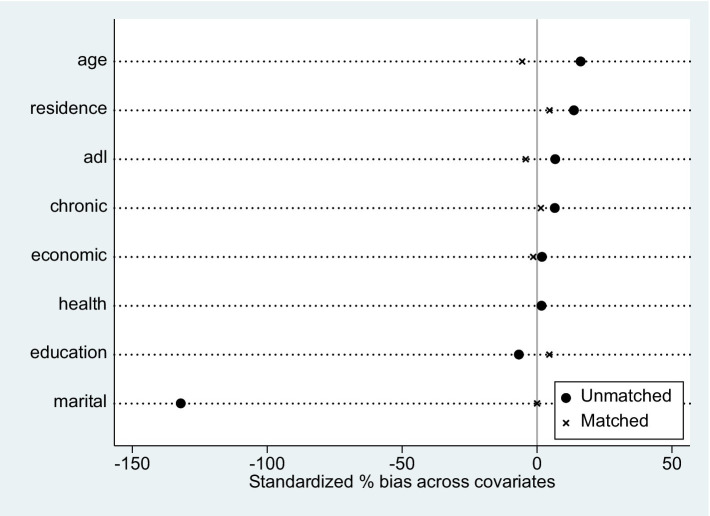
Distribution of control variables before and after matching.

At the same time, the matching effect diagram (see Figure 3) is generated to more intuitively visualize the distribution of covariate deviation between the treatment group and the control group before and after matching. After matching, there is a considerable reduction in the covariate variation across groups, indicating a satisfactory overall matching impact.

The average treatment effect is shown in Table 7. According to the findings, the net additional value of depressive symptoms in older adults caused by living alone is 1.745. After nuclear matching, ATT is 0.839, indicating a net added value of the depressive symptoms in older adults caused by living alone is 0.839. It may be considered that older people living alone will have much higher levels of depressive symptoms, which is consistent with the OLS regression model. The average processing impact (ATT) of nearest neighbor matching (k = 3) and radius matching is also noteworthy at the 1% statistical significance level, and the results obtained by the three matching methods have little difference, further confirming hypothesis 1.

**Table 7:** Propensity score matching results propensity score matching results.

Matching method	Treatment group	Control group	ATT	S. E.	*T*-stat
Before matching	10.548	8.803	1.745	0.201	8.67^***^
Nearest neighbor matching	10.548	9.679	0.870	0.306	2.84^***^
Radius matching	10.543	9.657	0.886	0.279	3.18^***^
Kernel matching	10.548	9.709	0.839	0.274	3.06^***^

***p < 0.001.

### 5.5 Mechanism analysis

The regression analysis results of social activity and adult children’s relationship satisfaction between living alone and depressive symptoms of older adults are shown in Table 8. Living alone is the independent variable in this study, while older adults’ depressive symptoms are the dependent variable. Social activity and adult children’s relationship satisfaction serve as intermediary variables. The control variables include gender, age, place of residence, education level, marital status, family economic position, self-rated health status, chronic diseases, and ADL. The results show that living alone can significantly positively predict social activity (*β* = 0.12, *p* < 0.001) and older adults’ depressive symptoms (*β* = 0.15, *p* < 0.001), and significantly negatively predict adult children’s relationship satisfaction (*β* = −0.12, *p* < 0.001). Older adults’ depressive symptoms were adversely predicted by adult children’s relationship satisfaction (*β* = −0.16, *p* < 0.001). Social activity significantly negatively predicted older adults’ depressive symptoms (*β* = −0.04, *p* < 0.001), but did not significantly predict adult children’s relationship satisfaction (*β* = −0.01, *p* > 0.05). That is to say, there is no chain mediation between social activity and adult children’s relationship satisfaction between living alone and older adults’ depressive symptoms. After accounting for intervening variables, it was found that living alone is associated with higher levels of depressive symptoms in older people (*β* = 0.13, *p* < 0.001). This suggests that social activity and adult children’s relationship satisfaction partially mediate the association between living alone and depressive symptoms of older adults.

**Table 8:** Regression model of mediating effects of social activity and child relationship satisfaction (standardized).

Variable	Depressive symptoms		Social activity		Adult children’s relationship satisfaction		Depressive symptoms	
	*β*	*t*	*β*	*t*	*β*	*t*	*β*	*t*
Living alone	0.15	4.56^***^	0.12	3.31^***^	−0.12	−3.32^***^	0.13	4.14^***^
Social activity					−0.01	−0.43	−0.04	−3.33^***^
Adult children’s relationship satisfaction							−0.16	−15.48^***^
R^2^	0.274		0.062		0.032		0.300	
F	251.444		43.726		19.909		238.166	

All models were tested controlling for gender, age, residence, education level, marital status, family economic position, self-rated health status, chronic illnesses, and ADL variables; ***p < 0.001.

Table 9 displays the outcomes of the chain mediation effect test conducted with Model 6 of the Process4.0 macro program. The total indirect effect value of social activity and adult children’s relationship satisfaction is 0.1011, accounting for 10.72% of the overall effect. There is no zero in the Bootstrap 95% confidence interval. The indirect effect value of living alone → social activity → depressive symptoms (path 1) is −0.0274, which is opposite to the sign of the direct effect value, indicating that social activity plays a “masking effect” between living alone and older adults’ depressive symptoms. The indirect effect value of living alone → adult children’s relationship satisfaction → depressive symptoms (path 2) is 0.1278, which represents 13.55% of the overall effect. The fact that there is no zero in the Bootstrap 95% confidence interval indicates that there is a mediating effect of adult children’s relationship satisfaction between living alone and older adults’ depressive symptoms. The indirect effect value of living alone → social activity → adult children’s relationship satisfaction → depressive symptoms (Path 3) is 0.0007, which represents 0.07% of the overall effect. The fact that there is no zero in the Bootstrap 95% confidence interval indicates that Path 3 is insignificant. This further confirms that there is no chained mediation effect in this mediation model.

**Table 9:** Chain mediation effect test.

Effect	Path	*β*	S. E.	95%CI		Mediation (%)
				LLCI	ULCI	
Direct effect	Direct path	0.8421	0.2034	0.4434	1.2408	89.28
Total indirect effect		0.1011	0.0422	0.0182	0.1845	10.72
Indirect effect	Path 1	−0.0274	0.0115	−0.0527	−0.0084	2.91
	Path 2	0.1278	0.0405	0.0492	0.2082	13.55
	Path 3	0.0007	0.0017	−0.0026	0.0042	0.07
Total effect		0.9432	0.2068	0.5378	1.3486	

LLCI and ULCI represent the lower limit and upper limit of the confidence interval, respectively. Path 1: living alone → social activity → depressive symptoms. Path 2: living alone → adult children’s relationship satisfaction → depressive symptoms. Path 3: living alone → social activity → adult children’s relationship satisfaction → depressive symptoms.

Table 10 displays the findings of testing the parallel mediation effect using Model 4 of the Process4.0 macro program. The values of the indirect effect of social activity and adult children’s relationship satisfaction were − 0.0274 and 0.1285. None of the Bootstrap 95% confidence intervals contained 0, indicating a significant mediation benefit. The outcomes of the parallel mediation model show that adult children’s relationship satisfaction has a stronger mediation effect than social activity. Specifically, the difference between the mediation effect of social activity and adult children’s relationship satisfaction is −0.1558. The Bootstrap 95% confidence interval is [−0.2429, −0.0729].

**Table 10:** Parallel mediation effect test.

Effect	Path	*β*	S. E.	95%CI		Mediation (%)
				LLCI	ULCI	
Direct effect	Direct path	0.8421	0.2034	0.4434	1.2408	89.28
Total indirect effect		0.1011	0.0423	0.0176	0.1882	10.72
Indirect effect	Path 1	−0.0274	0.0117	−0.0533	−0.0079	2.91
	Path 2	0.1285	0.0416	0.0474	0.2121	13.62
Total effect		0.9432	0.2068	0.5378	1.3486	

LLCI and ULCI represent the lower limit and upper limit of the confidence interval, respectively. Path 1: living alone → social activity → depressive symptoms. Path 2: living alone → adult children’s relationship satisfaction → depressive symptoms.

## 6 Discussion

### 6.1 Living alone has a dramatic positive influence on depressive symptoms in older people

Both the OLS regression model and PSM estimate results indicate that living alone is associated with higher levels of depressive symptoms in older adults, which is in accordance with the findings of earlier studies (21). On the one hand, older people who live alone are more susceptible to suffering feelings of isolation compared to adults who live with their relatives. Loneliness is a subjective phenomenon characterized by a detrimental emotional state driven by a mismatch between anticipated and actual social connections (52). The World Health Organization claims that more than a quarter of the global aged population experiences loneliness, leading to an elevated susceptibility to depression and maybe even suicide (53). According to a cross-sectional study conducted in Switzerland, loneliness had a positive link with depression and can both contribute to and result from the condition (54). A longitudinal study of aging in Britain also found that loneliness had a substantial association with the intensity of depressive symptoms in older adults after controlling for other confounding factors. Furthermore, this effect was shown to persist for a duration of 12 years. On the contrary, the level of depressive symptoms experienced by older adults who live alone is associated with the amount of social assistance they receive. Hayashi and other researchers regard living alone as an indicator of social vulnerability. Additionally, they also believed that social vulnerability might serve as an indicator of depressive symptoms (55). During the COVID-19 epidemic, older adults who live alone faced more challenges in accessing medical assistance compared to those who live with others. They also experienced a significant reduction in the social support they got and a constant increase in their levels of depressive symptoms (56). In addition, several scholars have demonstrated that cortisol and brain structure constitute significant factors in the development of severe depression. Older people who live alone are characterized by increased nighttime cortisol secretion and a more gradual day-night gradient. They exhibit higher levels of depressive symptoms (57). Thus, policymakers should prioritize the well-being of older people who live alone by collaborating with communities, universities, businesses, and other institutions. They should also establish regular care initiatives to provide economic assistance and emotional solace to these individuals. By building a monitoring system for older people living alone, the government can update their physical and mental health status in a timely manner, and systematically manage the occurrence and development of depression among older people living alone. Policymakers should start from the perspective of prevention to avoid the emergence of depression.

### 6.2 The association between living alone and depressive symptoms in older people is heterogeneous

The heterogeneity analysis results reveal significant disparities in the association of living alone with depressive symptoms in older people, depending on their age and geographical location. The results in Table 6 demonstrate that there are age differences in the association between living alone on depressive symptoms in older people. Young older people living alone experience a higher prevalence of depression and are at a greater risk of developing depression compared to older adults who live alone, which is consistent with earlier study findings (58). According to role theory, young older people who live alone are experiencing the transition of social roles, as well as the mental pressure of socioeconomic status and future planning. Consequently, they are more prone to experiencing negative emotions. The prevalence of living alone in older people has doubled or more, while in those aged 80 and above, living alone has no bearing on one’s likelihood of death (59). This could be attributed to the fact that as adults age, their experience and perspective tend to improve, enabling them to handle setbacks and pressures with composure. Additionally, older adults’ degree of depression significantly declines as their family life and finances become more secure. There are differences between urban and rural areas in the association of living alone on depressive symptoms in older people. Urban areas exhibit higher income and social welfare levels for older people living alone compared to rural areas. Additionally, urban areas boast superior medical service capacity and infrastructure development, leading to greater autonomy in the lives of these individuals (60). Nevertheless, older people living alone in rural areas frequently experience substandard living conditions and limited access to medical services as a result of poverty, inadequate transportation, and outdated healthcare facilities (61). In addition, since China’s rapid urbanization has led to a greater number of young people leaving rural areas in quest of employment opportunities, the proportion of older adult individuals living alone in rural areas has gradually increased. The disparities in income, social security systems, and infrastructure development between urban and rural regions have exacerbated the vulnerability of older people who live alone in rural areas to depression (47). The heterogeneity analysis reveals that living alone exerts a more pronounced association with the depressive symptoms of both young and older people residing in rural regions. Therefore, in forthcoming healthcare endeavors, policymakers should focus on older people who live alone, taking into account their distinct population characteristics, and implement more sophisticated preventive measures to promote active aging. Policymakers can address the issue of social role change among young older people living alone by creating employment opportunities in proximity to their residences. Additionally, they can establish medical assistance organizations in rural areas to improve access to healthcare services for older people living alone. This can be achieved by implementing medical records systems and offering personalized consultation services.

### 6.3 The mediating role of social activity between living alone and depressive symptoms of older people

The findings of the influencing mechanism indicate that living alone can be indirectly linked to depressive symptoms in older people through social activity. Furthermore, social activity moderates the association between living alone and the level of depressive symptoms experienced by the older population. On the one hand, living alone can be a positive predictor of social activity among older people, as supported by previous research findings (30). Optimal physical well-being is an essential prerequisite for older people to maintain independent living arrangements (62). According to the social choice theory, older people with physical and mental impairments will have limited opportunities to engage in social activities (29). Older adults who live alone possess greater physical strength and vitality, enabling them to engage in social activities more frequently, resulting in higher levels of social activity. Moreover, older people residing with their offspring experience a greater burden of household chores, resulting in diminished leisure time and vitality, consequently contributing to their comparatively limited engagement in social activities. On the other hand, social activity has a negative predictive effect on depressive symptoms in older people. Engaging in social activities can enhance older adults’ sense of belonging, alleviate the stress of daily life, enhance life satisfaction, and positively influence their physical and mental well-being (63). Activity theory promotes the regular engagement of older people in social activities that involve intellectual and physical exertion, rather than isolating them from all social responsibilities. Older people who actively participate in social activities are more likely to engage in communication with others and effectively manage negative emotions, resulting in a decrease in their levels of depressive symptoms. Consequently, policymakers can increase investment in community fitness equipment and establish dedicated spaces for older people, such as square dance venues and chess and card rooms. The community may create a high-quality social network by holding social activities such as sports meetings for older people and dumpling-making activities.

### 6.4 The mediating role of adult children’s relationship satisfaction between living alone and depressive symptoms of older people

The findings of the influencing mechanism indicate that living alone is associated with higher levels of depressive symptoms through adult children’s relationship satisfaction. Furthermore, adult children’s relationship satisfaction moderates the association between living alone and the level of depressive symptoms experienced by the older population. On the one hand, living alone has a negative correlation with adult children’s relationship satisfaction. The theory of intergenerational solidarity defines intergenerational relations as emotional cohesion between parents and adult children and realizes family unity through mutual support and reciprocity (64). Single older people receive less companionship and care from their offspring compared to older people who are not living alone. They experience emotional detachment, leading to a decrease in their adult children’s relationship (65). On the other hand, adult children’s relationship satisfaction also has a negative correlation with higher levels of depressive symptoms in older people (24). According to the theory of separation, with the growth of age, the physical function and social scope of older people steadily deteriorate. They are no longer suitable for social responsibilities. Conversely, older adults have a heightened requirement for emotional support and a strong inclination to cultivate more intimate connections with others. At this time, older people rely primarily on family support and companionship as their main source of emotional nourishment. A longitudinal study shows that close intergenerational relationships might lessen the negative effects of traumatic experiences and ease the psychological pressure that comes with older adults living alone (66). Older people who experience greater satisfaction with their adult children’s relationships tend to engage in more frequent meetings or communication with their adult children. For example, older adults who feel isolated will experience reduced feelings of loneliness and receive increased emotional support through video and WeChat conversations. This will foster a sense of belonging and self-worth, ultimately leading to a decrease in their levels of depressive symptoms (67). Hence, it is imperative for adult children to not only focus on providing material assistance to older people but also prioritize offering spiritual support. By engaging in direct or online interactions, individuals should enhance their communication with older people, providing them with timely support and attention. This will foster a strong intergenerational bond and help mitigate the likelihood of depression.

## 7 Limitation

There were also some limitations in this study: Firstly, the cross-sectional data from CHARLS 2020 were used. Even with such a large and diverse sample size, it was challenging to dynamically represent the association between living alone and depressive symptoms in older adults. Secondly, the cross-sectional study design could not rule out reverse causation, especially when a percentage of the sample would have crossed the threshold of the CES-D to warrant a depression diagnosis. In the future, we can examine the association between living alone and depressive symptoms of older people through longitudinal studies.

## 8 Summary

This study examines the association between living alone and depression in older people using data from the 2020 China Health and Retirement Longitudinal Study. The study also considers the mediating role of social activity and adult children’s relationship satisfaction. The findings indicate that living alone is associated with older people’s higher levels of depressive symptoms. This association is stronger in older adults aged 60–74 or rural areas. The link between living alone and depressive symptoms of older people is mediated by both social activity and adult children’s relationship satisfaction. This has immense importance in elucidating the association between living alone and depressive symptoms of older people. Additionally, it offers insights on how to avoid depression in older people, hence minimizing the associated risk.

## Data Availability

The original contributions presented in the study are included in the article/Supplementary material, further inquiries can be directed to the corresponding authors.
