# Supplementary material for: The association between living alone and depressive symptoms in older adults population: evidence from the China Health and Retirement Longitudinal Study
Source: Front Public Health. 2024 Oct 9;12:1441006. doi: 10.3389/fpubh.2024.1441006 (PMC11496203; doi:10.3389/fpubh.2024.1441006)
Supplement: Supplementary file 1 [file Table_1.DOCX]

Supplementary Material. The matching test results of nearest neighbor matching (k=3) and radius matching.

Table1 Balance test: nearest neighbor matching (k=3)

| Variable | Sample | Mean | | *S. E* | %bias | T-test | |
| --- | --- | --- | --- | --- | --- | --- | --- |
|  |  | Treated | Control |  |  | *t* | *p* |
| Age | U | 69.731 | 68.671 | 16.1 | 84.3 | 5.46 | 0.000 |
|  | M | 69.731 | 69.564 | 2.5 |  | 0.59 | 0.553 |
| Residence | U | 0.663 | 0.597 | 13.7 | 80.3 | 4.35 | 0.000 |
|  | M | 0.663 | 0.650 | 2.7 |  | 0.69 | 0.489 |
| Educational level | U | 1.910 | 1.983 | -6.7 | 84.7 | -2.17 | 0.030 |
|  | M | 1.910 | 1.922 | -1.0 |  | -0.26 | 0.793 |
| Marital status | U | 0.405 | 0.925 | -132.1 | 100.0 | -52.42 | 0.000 |
|  | M | 0.405 | 0.405 | 0.0 |  | 0.00 | 1.000 |
| family economic status | U | 9.429 | 9.412 | 1.8 | -77.2 | 0.62 | 0.534 |
|  | M | 9.429 | 9.459 | -3.2 |  | -0.80 | 0.423 |
| Self-reported health status | U | 2.995 | 2.979 | 1.7 | -0.5 | 0.53 | 0.593 |
|  | M | 2.995 | 2.979 | 1.7 |  | 0.41 | 0.680 |
| Chronic diseases | U | 0.882 | 0.860 | 6.6 | 85.9 | 2.07 | 0.038 |
|  | M | 0.882 | 0.879 | 0.9 |  | 0.24 | 0.808 |
| ADL | U | 0.562 | 0.487 | 6.7 | 66.6 | 2.21 | 0.027 |
|  | M | 0.562 | 0.587 | -2.2 |  | -0.54 | 0.591 |

Source: Author’s calculation using 2020 China Health and Retirement Longitudinal Study data of 6,688 eligible study participants.

*Note*: ″U″ represent the unmatched subsample, and ″M″ represent the matched subsample; % bias denotes mean standardized difference in percentage; ***P<0.001, **P<0.01, *P<0.05.

Table2 Balance test: radius matching (r=0.01)

| Variable | Sample | Mean | | *S. E* | %bias | T-test | |
| --- | --- | --- | --- | --- | --- | --- | --- |
|  |  | Treated | Control |  |  | *t* | *p* |
| Age | U | 69.731 | 68.671 | 16.1 | 98.4 | 5.46 | 0.000 |
|  | M | 69.714 | 69.731 | -0.3 |  | -0.06 | 0.950 |
| Residence | U | 0.663 | 0.597 | 13.7 | 88.6 | 4.35 | 0.000 |
|  | M | 0.663 | 0.655 | 1.6 |  | 0.40 | 0.689 |
| Educational level | U | 1.910 | 1.983 | -6.7 | 76.6 | -2.17 | 0.030 |
|  | M | 1.909 | 1.926 | -1.6 |  | -0.40 | 0.690 |
| Marital status | U | 0.405 | 0.925 | -132.1 | 100.0 | -52.42 | 0.000 |
|  | M | 0.406 | 0.406 | -0.0 |  | 0.00 | 1.000 |
| family economic status | U | 9.429 | 9.412 | 1.8 | -23.7 | 0.62 | 0.534 |
|  | M | 9.434 | 9.455 | -2.3 |  | -0.58 | 0.563 |
| Self-reported health status | U | 2.995 | 2.979 | 1.7 | 67.1 | 0.53 | 0.593 |
|  | M | 2.998 | 2.992 | 0.5 |  | 0.13 | 0.893 |
| Chronic diseases | U | 0.882 | 0.860 | 6.6 | 89.7 | 2.07 | 0.038 |
|  | M | 0.882 | 0.884 | -0.7 |  | -0.18 | 0.858 |
| ADL | U | 0.562 | 0.487 | 6.7 | 52.3 | 2.21 | 0.027 |
|  | M | 0.555 | 0.591 | -3.2 |  | -0.77 | 0.444 |

Source: Author’s calculation using 2020 China Health and Retirement Longitudinal Study data of 6,688 eligible study participants.

*Note*: ″U″ represent the unmatched subsample, and ″M″ represent the matched subsample; % bias denotes mean standardized difference in percentage; ***P<0.001, **P<0.01, *P<0.05.
